# Gliomas: Application of Cumulative Histogram Analysis of Normalized Cerebral Blood Volume on 3 T MRI to Tumor Grading

**DOI:** 10.1371/journal.pone.0063462

**Published:** 2013-05-21

**Authors:** Hyungjin Kim, Seung Hong Choi, Ji-Hoon Kim, Inseon Ryoo, Soo Chin Kim, Jeong A. Yeom, Hwaseon Shin, Seung Chai Jung, A. Leum Lee, Tae Jin Yun, Chul-Kee Park, Chul-Ho Sohn, Sung-Hye Park

**Affiliations:** 1 Department of Radiology, Seoul National University College of Medicine, Seoul, Korea; 2 Department of Neurosurgery, Seoul National University College of Medicine, Seoul, Korea; 3 Department of Pathology, Seoul National University College of Medicine, Seoul, Korea; 4 Center for Nanoparticle Research, Institute for Basic Science, and School of Chemical and Biological Engineering, Seoul National University, Seoul, Korea; Instituto de Investigación Sanitaria INCLIVA, Spain

## Abstract

**Background:**

Glioma grading assumes significant importance in that low- and high-grade gliomas display different prognoses and are treated with dissimilar therapeutic strategies. The objective of our study was to retrospectively assess the usefulness of a cumulative normalized cerebral blood volume (nCBV) histogram for glioma grading based on 3 T MRI.

**Methods:**

From February 2010 to April 2012, 63 patients with astrocytic tumors underwent 3 T MRI with dynamic susceptibility contrast perfusion-weighted imaging. Regions of interest containing the entire tumor volume were drawn on every section of the co-registered relative CBV (rCBV) maps and T2-weighted images. The percentile values from the cumulative nCBV histograms and the other histogram parameters were correlated with tumor grades. Cochran’s Q test and the McNemar test were used to compare the diagnostic accuracies of the histogram parameters after the receiver operating characteristic curve analysis. Using the parameter offering the highest diagnostic accuracy, a validation process was performed with an independent test set of nine patients.

**Results:**

The 99th percentile of the cumulative nCBV histogram (nCBV C99), mean and peak height differed significantly between low- and high-grade gliomas (P = <0.001, 0.014 and <0.001, respectively) and between grade III and IV gliomas (P = <0.001, 0.001 and <0.001, respectively). The diagnostic accuracy of nCBV C99 was significantly higher than that of the mean nCBV (P = 0.016) in distinguishing high- from low-grade gliomas and was comparable to that of the peak height (P = 1.000). Validation using the two cutoff values of nCBV C99 achieved a diagnostic accuracy of 66.7% (6/9) for the separation of all three glioma grades.

**Conclusion:**

Cumulative histogram analysis of nCBV using 3 T MRI can be a useful method for preoperative glioma grading. The nCBV C99 value is helpful in distinguishing high- from low-grade gliomas and grade IV from III gliomas.

## Introduction

Gliomas, which are tumors derived from glial cells, are the most common primary malignant brain neoplasms [1] and the incidence of gliomas has increased worldwide since the late 1970s [2]. Malignant gliomas are classified in the World Health Organization (WHO) system into four grades by the presence of certain tumor characteristics: atypia, mitoses, endothelial proliferation and necrosis [3]. Glioma grading is of the utmost importance because high-grade gliomas exhibit dismal prognoses and are usually treated with adjuvant radiation therapy or chemotherapy after resection, whereas low-grade gliomas are not [4].

Currently, magnetic resonance imaging (MRI) plays a crucial role in preoperative glioma grading and several methods, including MR spectroscopy, diffusion and perfusion imaging, have been used to differentiate high- from low-grade gliomas. A few recent reports have suggested that high-grade gliomas exhibit lower apparent diffusion coefficients (ADCs) and higher cerebral blood volumes (CBVs) in the solid malignant portion [4]–[13]. These results are plausible because high-grade tumors exhibit increased cellularity and vascularity with new vessels and damaged mature vessels [4], [7]. However, the evaluation of gliomas based on a few regions of interest (ROIs) placed on a representative section of the tumor is inherently limited, as this method, the so-called hot-spot method, is user-dependent and prone to sampling error [14], which may consequently lead to tumor under-grading. A potential solution for this disadvantage of conventional ROI analysis is a histogram method, which covers all of the tumor voxel data.

To date, there have only been a few published reports regarding glioma grading using a histogram analysis of imaging data [6], [8], [11]–[17]. Recently, Kang et al. [8] revealed that the 5^th^ percentile of the cumulative ADC histogram obtained at a high b-value diffusion-weighted imaging (DWI) was the most promising parameter for differentiating high- from low-grade gliomas. In terms of perfusion-weighted imaging (PWI), Emblem et al. [6] demonstrated that a histogram analysis of normalized CBV (nCBV) heterogeneity offered higher sensitivity, equal specificity and increased interobserver agreement compared with the hot-spot method in glioma grading.

To the best of our knowledge, there have been no previous reports examining the cumulative nCBV histogram parameters for the differentiation of glioma grades. Thus, the purpose of our study was to identify the cumulative nCBV histogram parameter with the best diagnostic accuracy in glioma grading using a 3 T MRI system.

## Materials and Methods

This retrospective study was approved by the institutional review board of Seoul National University Hospital. The institutional review board waived the need for written informed consent from the participants.

### Patient Selection

From February 2010 to April 2012, one author (S.H.C.) retrospectively reviewed the electronic medical records and the radiology information systems of our hospital for patients with astrocytic tumors. The inclusion criteria were as follows: (a) a histopathologic diagnosis of astrocytic tumors without oligodendroglial components according to the WHO criteria and (b) 3 T MR imaging with dynamic susceptibility contrast (DSC) PWI prior to surgery or chemoradiotherapy. We excluded 5 patients due to the following reasons: (a) inadequate MR imaging quality due to substantial motion or susceptibility artifacts (n = 4) and (b) the absence of PWI raw data (n = 1).

**Figure 1:**
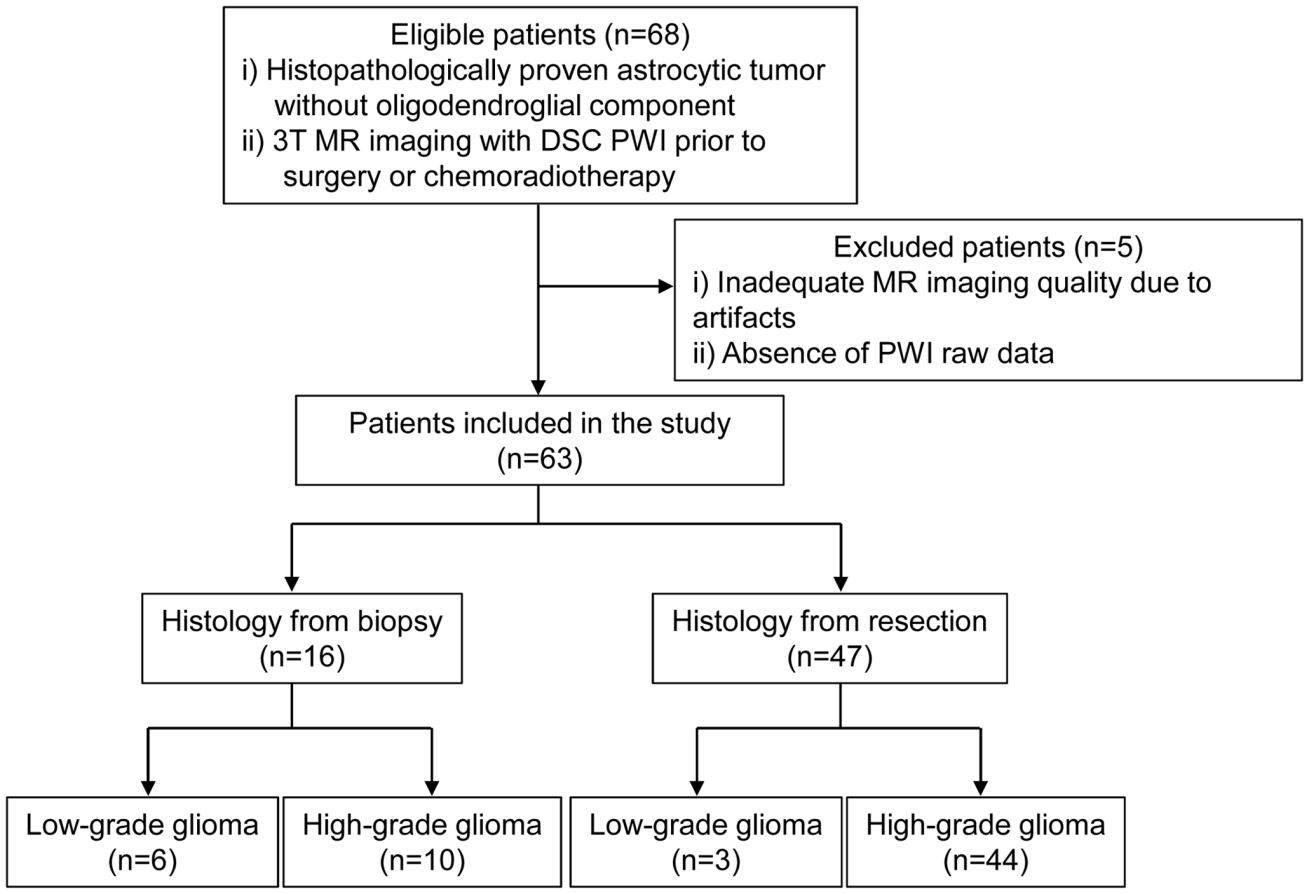
Flow diagram of patient selection and inclusion and exclusion criteria. DWI = diffusion-weighted imaging; DSC = dynamic susceptibility contrast; PWI = perfusion-weighted imaging.

A total of 63 patients were included in the study. Among the 63 enrolled patients, 9 exhibited WHO grade II astrocytomas, 16 exhibited WHO grade III astrocytomas and 38 exhibited WHO grade IV glioblastomas (Fig. 1). Grade III astrocytomas and grade IV glioblastomas were classified as high-grade gliomas, while grade II astrocytomas were categorized as low-grade gliomas.

We enrolled an additional nine patients for an independent test set. These patients had undergone surgical resection in our institution between May 2012 and December 2012 and met the inclusion criteria described above.

### Image Acquisition

All MRI examinations were performed with a single 3 T MR system (Verio; Siemens Medical Solutions, Erlangen, Germany) with a 32-channel head coil. The brain imaging sequences included axial spin-echo T1-weighted (T1W) images, fast spin-echo T2-weighted (T2W) images, fluid-attenuated inversion-recovery (FLAIR) images, DSC PWI with gadobutrol (Gadovist, Bayer Schering Pharma, Berlin, Germany) and subsequent contrast-enhanced spin-echo T1W images. The MR imaging parameters were as follows: 558/9.8 ms/70°/384×212 (TR/TE/FA/matrix) for spin-echo T1W images, 5160/91 ms/130°/640×348 (TR/TE/FA/matrix) for fast spin-echo T2W images and 9,000/97 ms/130°/384×209 (TR/TE/FA/matrix) for FLAIR images. The other parameters for the three imaging sequences were as follows: section thickness, 5 mm with a 1-mm gap and a field of view (FOV) of 240×240 mm.

For DSC PWI, a single-shot gradient-echo EPI sequence was used for imaging during the intravenous injection of the contrast material. The imaging parameters of DSC PWI were as follows: TR/TE, 1500/30 ms; FA, 90°; FOV, 240×240 mm; 16 sections; matrix, 128×128; section thickness, 5 mm; intersection gap, 1 mm; and voxel resolution, 1.86×1.86×5 mm. For each section, 60 images were obtained at intervals equal to the repetition time. After four to five time points, a bolus of gadobutrol at a dose of 0.1 mmol/kg of body weight and a rate of 4 mL/sec was injected with an MR-compatible power injector (Spectris; Medrad, Pittsburgh, PA, USA).

### Post-processing and Histogram Analysis

The MR imaging data for the conventional MR images and the DSC PWI were first transferred from the picture archiving and communication system workstation to a personal computer for further analyses. The relative CBV (rCBV) was acquired using a dedicated software package (nordicICE; NordicNeuroLab AS, Bergen, Norway) with an established tracer kinetic model applied to the first-pass data [18], [19]. First, realignment was performed to minimize patient motion during the dynamic scans. A gamma-variate function, which approximates the first-pass response as it would appear in the absence of recirculation, was used to fit the 1/T2* curves to reduce the effects of recirculation. To reduce contrast agent leakage effects, the dynamic curves were mathematically corrected [20]. After the elimination of recirculation and leakage of the contrast agent, the rCBV was computed by means of numeric integration of the curve. To minimize variances in the rCBV value in an individual patient, the pixel-based rCBV maps were normalized by dividing every rCBV value in a specific section by the rCBV value in the contralateral mirror-image unaffected white matter as defined by a neuroradiologist (S.H.C.) [21]. Co-registrations between the T2W images and rCBV maps were performed by using a dedicated software package (nordicICE; NordicNeuroLab AS, Bergen, Norway) [22].

Next, two observers (H.K. and J.H.K.) who were blinded to the clinical data drew ROIs in consensus that contained the entire tumor on every continuous sections of the co-registered images. Tumor boundaries were defined with reference to the high-signal intensity areas thought to represent tumor tissue on the T2W images [8]. Areas of necrosis, hemorrhage, or macro-vessels were first identified on the conventional MR imaging sequences and were excluded carefully from the ROIs as described in previous studies [6], [12], [16], [20], [23]. After obtaining the total voxel values of the nCBV of each tumor, histograms were plotted.

For the cumulative nCBV histogram, the cumulative number of observations in all of the bins up to the specified bin was mapped on the y-axis as a percentage with nCBV on the x-axis. The percentile values from the 70^th^ to the 100^th^ percentile were derived from the cumulative nCBV histogram (the Xth percentile value is the point at which X% of the voxel values that form the histogram are found to the left of the histogram) [8], [14]. We took into account the caveats that the voxel values at both extreme ends of a cumulative histogram are prone to image noise and other sources of false voxel values (e.g., spikes introduced by the algorithms used to generate the rCBV maps) [6]. Nevertheless, the 100^th^ percentile value was included as this value was identical to the maximum nCBV.

As for the nCBV histogram, the area under the histogram curve was normalized to the value of one and the number of histogram bins was 108 as suggested by Emblem et al. [6]. The following histogram parameters were also obtained: (a) mean; (b) standard deviation (SD); (c) mode, which equals the peak height position; (d) kurtosis, which is the degree of peakedness of the distribution; (e) skewness, which is the measure of the degree of asymmetry of the distribution; and (f) peak height, which is the relative frequency of nCBV in a given histogram bin.

### Statistical Analysis

All statistical analyses were performed using two commercial software programs (MedCalc version 12.3.0, MedCalc Software, Mariakerke, Belgium; and SPSS version 18.0, SPSS Inc., Chicago, Illinois). A P-value <0.05 was considered statistically significant.

To identify the percentile value of the cumulative nCBV histogram with highest diagnostic accuracy for differentiating high- from low-grade gliomas, the areas under the receiver operating characteristic (ROC) curves (AUCs) were obtained for each percentile value. From the ROC curve analysis for the optimum cumulative histogram parameter, the cutoff value with the best sensitivity and specificity was obtained.

Kolmogorov-Smirnov’s test was used to determine whether the non-categorical variables of the histogram parameters were normally distributed. According to the results of the Kolmogorov-Smirnov’s test, an unpaired Student t-test or Mann-Whitney U-test was performed, as appropriate, to compare the histogram parameters between the low- and high-grade gliomas. A one-way analysis of variance (ANOVA) or the Kruskal-Wallis test was performed for the comparison of histogram parameters among WHO grade II, III and IV gliomas. After obtaining cutoff values, sensitivities and specificities using the ROC curve analysis, Cochran’s Q test was used to compare the diagnostic accuracies among the multiple parameters. The pairwise McNemar test was subsequently performed. For the pairwise comparisons among the three tumor grades and among the three different parameters, the Bonferroni correction was applied to minimize α (i.e., type I) error [24]. The significance level (P = 0.05) was therefore reduced to an α-adjusted P level of 0.017.

After determining the parameter demonstrating the highest diagnostic accuracy obtained through the above analyses, we planned to suggest a potential diagnostic algorithm that can differentiate the three different WHO glioma grades. The cutoff values of the parameter were subsequently applied to the test set.

## Results

**Figure 2:**
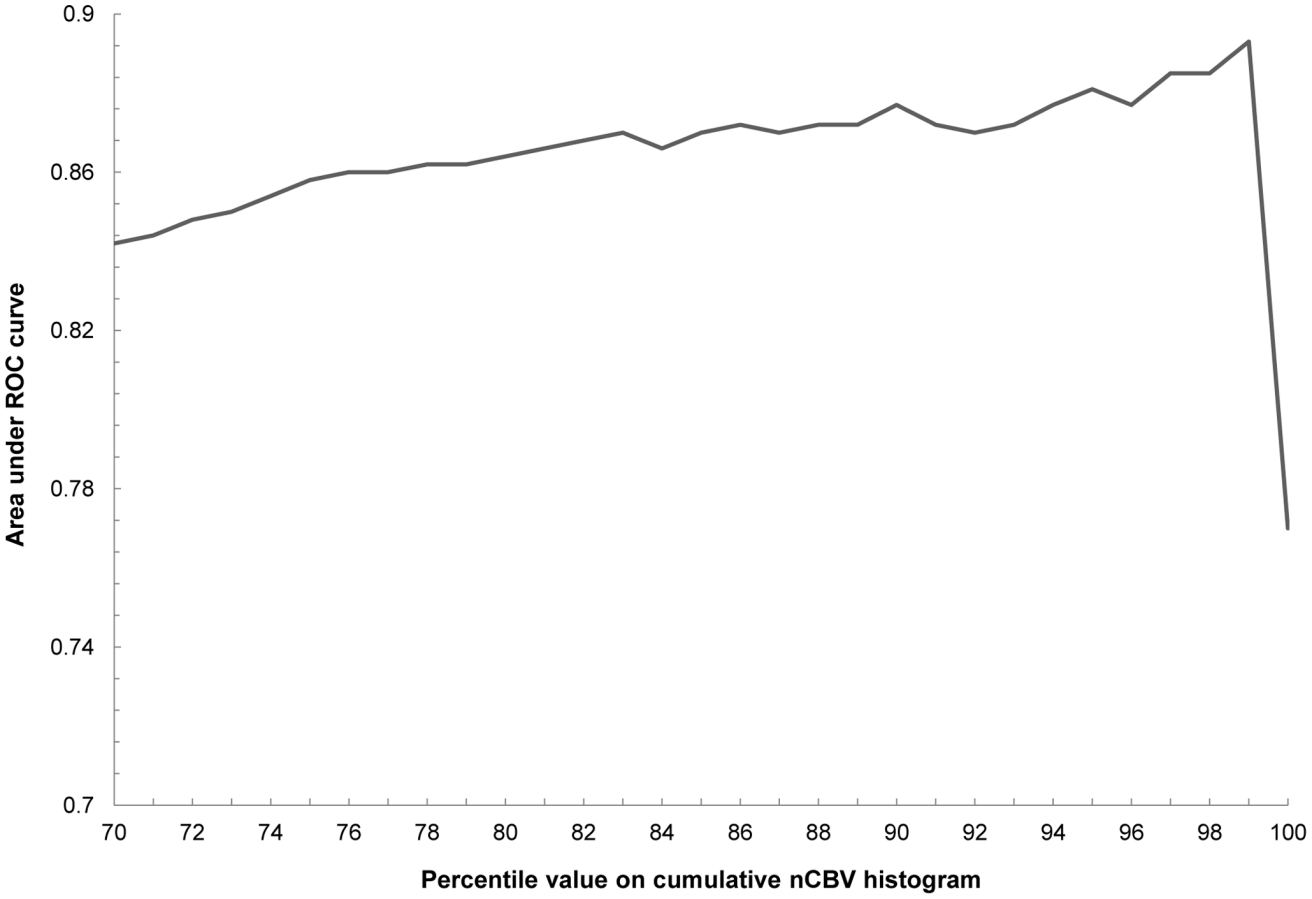
The area under the ROC curve is displayed on the y-axis according to each percentile of the cumulative nCBV histogram on the x-axis. The 99^th^ percentile value exhibited the highest AUC (0.893) for differentiating high- from low-grade gliomas.

Of the 70^th^ to the 100^th^ percentile values of the cumulative nCBV histogram, the nCBV C99 (the 99^th^ percentile) exhibited the highest AUC (0.893) for differentiating high- from low-grade gliomas (Fig. 2).

**Figure 3:**
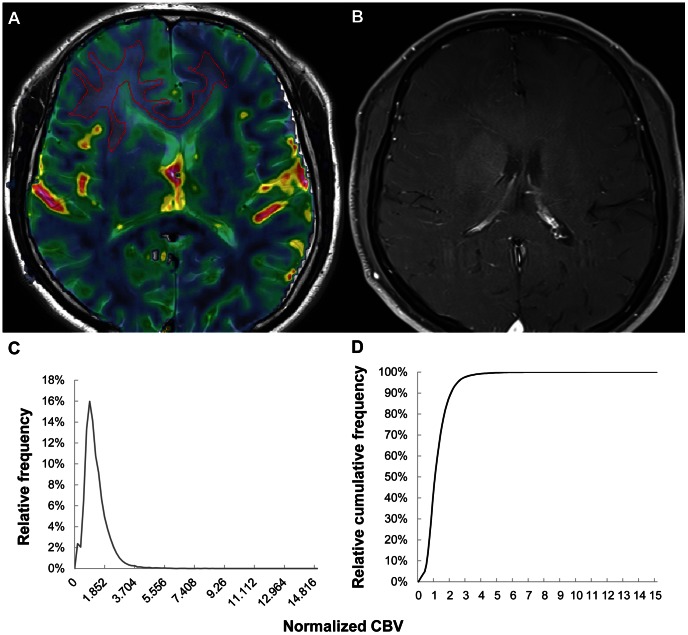
Images of a 55-year-old male with a grade II astrocytoma. (A) ROI-drawn rCBV map overlaid on a T2-weighted image, (B) contrast-enhanced T1-weighted image, (C) histogram of nCBV and (D) cumulative nCBV histogram. The 99^th^ percentile value of the cumulative nCBV histogram was 3.703. ROI = region of interest; rCBV = relative CBV; nCBV = normalized CBV.

**Figure 4:**
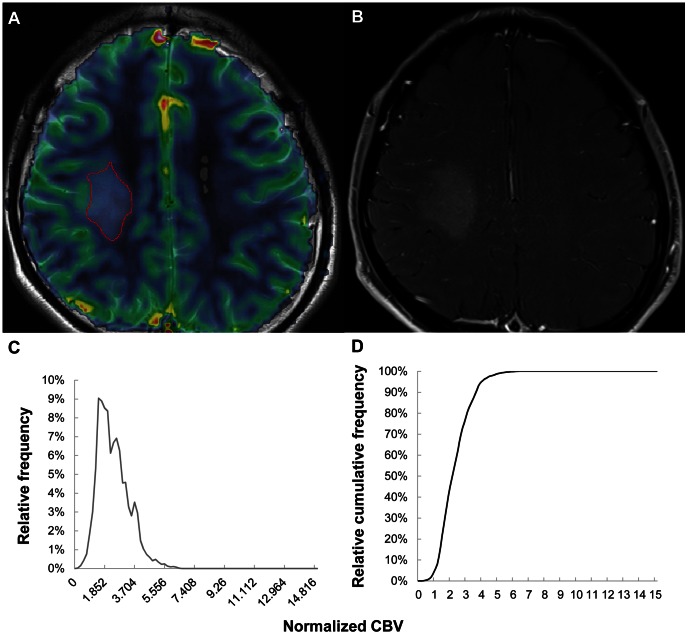
Images of a 35-year-old male with a grade III astrocytoma. (A) ROI-drawn rCBV map overlaid on a T2-weighted image, (B) contrast-enhanced T1-weighted image, (C) histogram of nCBV and (D) cumulative nCBV histogram. The 99^th^ percentile value of the cumulative nCBV histogram was 5.092. ROI = region of interest; rCBV = relative CBV; nCBV = normalized CBV.

**Figure 5:**
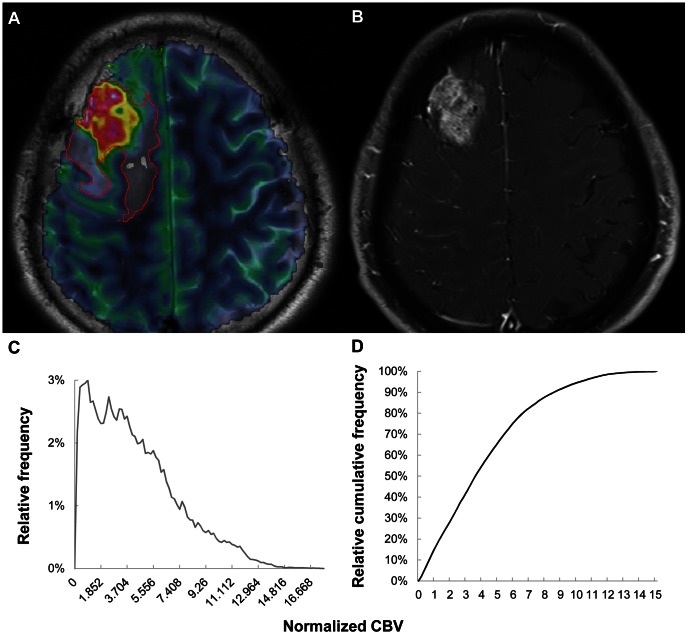
Images of a 50-year-old female with a grade IV glioblastoma. (A) ROI-drawn rCBV map overlaid on a T2-weighted image, (B) contrast-enhanced T1-weighted image, (C) histogram of nCBV and (D) cumulative nCBV histogram. The 99^th^ percentile value of the cumulative nCBV histogram was 12.489. ROI = region of interest; rCBV = relative CBV; nCBV = normalized CBV.

In the comparisons of multiple histogram parameters between low- and high-grade gliomas (Table 1), the nCBV C99, mean and peak height were significantly different between grades (P = <0.001, 0.014 and <0.001, respectively). No significant difference was found between low- and high-grade gliomas with respect to mode, kurtosis and skewness (P = 0.953, 0.398 and 0.492, respectively). Next, glioma grades II, III and IV were separated and compared with an ANOVA or the Kruskal-Wallis test (Table 2). The nCBV C99, mean and peak height differed significantly between grades II and IV (P = <0.001, 0.002 and <0.001, respectively) and between grades III and IV (P = <0.001, 0.001 and <0.001, respectively). However, no significant difference was observed among the three different grades with respect to mode, kurtosis and skewness (P = 0.493, 0.680 and 0.785, respectively). Figures 3, 4 and 5 present the representative cases of grade II, III and IV gliomas, respectively, with rCBV maps and cumulative nCBV histograms.

**Table 1:** nCBV histogram parameters of low- and high-grade gliomas.

Parameter	Low-grade glioma (n = 9)	High-grade glioma (n = 54)	P-value*
nCBV C99	3.55±0.55	8.72±6.79	<0.001
Mean	1.35±0.27	2.59±1.46	0.014
Mode	0.88±0.51	1.17±1.04	0.953
Kurtosis	6.16±7.80	8.43±18.94	0.398
Skewness	1.62±0.91	1.65±1.48	0.492
Peak height	0.21±0.06	0.11±0.06	<0.001

Note − Unless otherwise specified, the data are the mean ± standard deviations.

* P-values for the comparison of the mean and peak height were calculated using the unpaired Student t-test and other P-values were calculated using the Mann-Whitney U-test.

nCBV = normalized CBV; nCBV C99 = 99^th^ percentile value on the cumulative nCBV histogram.

**Table 2:** nCBV histogram parameters of the three different WHO glioma grades.

				P-value*		
Parameter	Grade II (n = 9)	Grade III (n = 16)	Grade IV (n = 38)	Grade II vs. III	Grade II vs. IV	Grade III vs. IV
nCBV C99	3.55±0.55	4.16±1.42	10.64±7.25	1	<0.001	<0.001
Mean	1.35±0.27	1.58±0.65	3.02±1.50	0.910	0.002	0.001
Mode	0.88±0.51	1.12±0.62	1.20±1.17	0.493		
Kurtosis	6.16±7.80	5.84±13.03	9.52±21.00	0.680		
Skewness	1.62±0.91	1.49±1.18	1.71±1.59	0.785		
Peak height	0.21±0.06	0.16±0.07	0.08±0.04	0.090	<0.001	<0.001

Note − Unless otherwise specified, the data are the means ± standard deviations.

* P-values for the comparison of the mean and peak height were calculated using a one-way analysis of variance and the other P-values were calculated using the Kruskal-Wallis test.

nCBV = normalized CBV; nCBV C99 = 99^th^ percentile value on cumulative nCBV histogram.

Table 3 summarizes the results of the ROC analyses of the histogram parameters used to distinguish high- from low-grade gliomas. The nCBV C99 cutoff value of 4.681 exhibited a sensitivity, specificity and accuracy of 85.2% (46/54), 100% (9/9) and 87.3% (55/63), respectively. The mean nCBV cutoff value of 1.786 exhibited sensitivity, specificity and accuracy values of 72.2% (39/54), 100% (9/9) and 76.2% (48/63), respectively. The peak height cutoff value of 0.150 exhibited sensitivity, specificity and accuracy values of 83.3% (45/54), 100% (9/9) and 85.7% (54/63), respectively. There was a significant difference among the diagnostic accuracies of the nCBV C99, mean and peak height (Cochran’s Q = 12.286, P = 0.002). When pairwise comparisons were performed, the diagnostic accuracy of the nCBV C99 was significantly higher than that of the mean nCBV on the McNemar test (P = 0.016). However, there were no significant differences between the nCBV C99 and peak height (P = 1.000) and between the mean and peak height (P = 0.031).

**Table 3:** ROC results for nCBV histogram parameters for glioma grading (low- vs. high-grade).

	nCBV C99	Mean nCBV	Peak height
AUC*	0.893 (0.789, 0.957)	0.844 (0.730, 0.923)	0.893 (0.789, 0.957)
Sensitivity (%)†	85.2 (46/54)	72.2 (39/54)	83.3 (45/54)
Specificity (%)†	100 (9/9)	100 (9/9)	100 (9/9)
Accuracy (%)†	87.3 (55/63)	76.2 (48/63)	85.7 (54/63)
Cutoff Value	>4.681	>1.786	≤0.150
P-value for ROC curve	<0.0001	<0.0001	<0.0001
P-value for Cochran’s Q test of diagnostic accuracy	0.002		

Note − *Data in parentheses are 95% confidence intervals.

† Sensitivity, specificity and accuracy for identifying high-grade gliomas. The data in parentheses are the numbers used to calculate the percentages.

nCBV = normalized CBV; nCBV C99 = 99^th^ percentile value on cumulative nCBV histogram; AUC = area under the ROC curve; ROC = receiver operating characteristic.

We subsequently performed ROC curve analyses of the nCBV C99, mean and peak height for the differentiation between grade III and IV gliomas (Table 4). The nCBV C99 cutoff value of 6.335 exhibited sensitivity, specificity and accuracy values of 84.2% (32/38), 100% (16/16) and 88.9% (48/54), respectively. The mean nCBV cutoff value of 2.304 exhibited sensitivity, specificity and accuracy values of 68.4% (26/38), 87.5% (14/16) and 74.1% (40/54), respectively. The peak height cutoff value of 0.105 exhibited sensitivity, specificity and accuracy values of 81.6% (31/38), 75.0% (12/16) and 79.6% (43/54), respectively. There was a significant difference among the diagnostic accuracies of the nCBV C99, mean and peak height (Cochran’s Q = 6.125, P = 0.047). However, when pairwise comparisons were performed with an α-adjusted P level of 0.017, no significant differences were found between the nCBV C99 and mean (P = 0.021), between the nCBV C99 and peak height (P = 0.227) and between the mean and peak height (P = 0.549).

**Table 4:** ROC results for nCBV histogram parameters for glioma grading (Grade III vs. IV).

	nCBV C99	Mean nCBV	Peak height
AUC*	0.974 (0.889, 0.998)	0.850 (0.727, 0.933)	0.829 (0.702, 0.918)
Sensitivity (%)†	84.2 (32/38)	68.4 (26/38)	81.6 (31/38)
Specificity (%)†	100 (16/16)	87.5 (14/16)	75.0 (12/16)
Accuracy (%)†	88.9 (48/54)	74.1 (40/54)	79.6 (43/54)
Cutoff Value	>6.335	>2.304	≤0.105
P-value for ROC curve	<0.0001	<0.0001	<0.0001
P-value for Cochran’s Q test of diagnostic accuracy	0.047		

Note − *Data in parentheses are 95% confidence intervals.

† Sensitivity, specificity and accuracy for identifying grade IV gliomas. The data in parentheses are the numbers used to calculate the percentages.

nCBV = normalized CBV; nCBV C99 = 99^th^ percentile value on cumulative nCBV histogram; AUC = area under the ROC curve; ROC = receiver operating characteristic.

**Figure 6:**
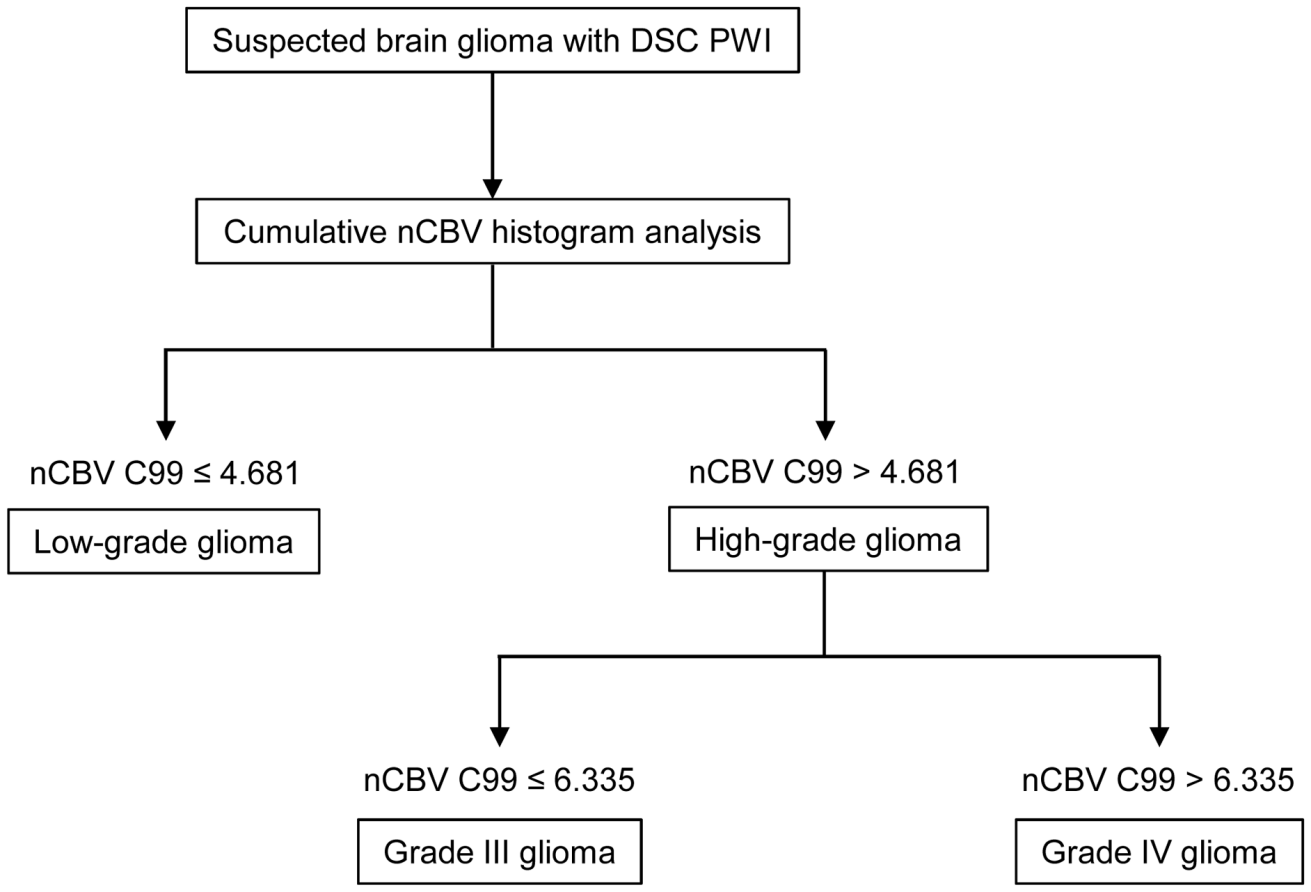
Flow diagram explaining the potential grading schema of gliomas using the cumulative nCBV histogram parameter. The nCBV C99 cutoff value of 4.681 was used to differentiate high- from low-grade gliomas and the nCBV C99 cutoff value of 6.335 was used to identify grade IV gliomas. nCBV = normalized CBV; nCBV C99 = 99^th^ percentile value of nCBV cumulative histogram.

As nCBV C99 displayed the highest diagnostic accuracy for differentiating high- from low-grade gliomas and grade IV from III gliomas, we designed a potential diagnostic algorithm with two cutoff values of nCBV C99 (Fig. 6). The nCBV C99 cutoff value of 4.681 was used to differentiate high- from low-grade gliomas and the nCBV C99 cutoff value of 6.335 was used to identify grade IV gliomas. These two values were applied to the test set of nine patients. Out of the nine total patients, we could diagnose grade II gliomas with 100% (2/2) diagnostic accuracy, grade III gliomas with 33.3% (1/3) diagnostic accuracy and grade IV glioblastomas with 75% (3/4) diagnostic accuracy (Table 5).

**Table 5:** Application of the glioma grading schema to the test set of nine patients.

Patient number	WHO glioma grade	nCBV C99	Diagnosis*
1	II	4.335	Correct
2	II	4.032	Correct
3	III	5.268	Correct
4	III	6.831	Incorrect
5	III	2.330	Incorrect
6	IV	5.083	Incorrect
7	IV	7.665	Correct
8	IV	6.664	Correct
9	IV	10.626	Correct

Note − *Diagnosis according to the suggested glioma grading schema in Figure 6 using the two nCBV C99 cutoff values to separate the three different WHO glioma grades.

nCBV = normalized CBV; nCBV C99 = 99^th^ percentile value on cumulative nCBV histogram.

## Discussion

The present study regarding glioma grading with histogram parameters derived from rCBV maps demonstrated that the nCBV C99, mean and peak height differed significantly between low- and high-grade gliomas and between grade III and IV gliomas. The diagnostic accuracy of the nCBV C99 was significantly higher than that of the mean nCBV in distinguishing high- from low-grade gliomas and was comparable to that of the peak height.

There have been many reports of glioma grading using PWI, although only a few studies have dealt with the histogram method to date [6], [11]–[13], [16]. Most previous studies using a conventional ROI analysis placed only three to five ROIs on the representative solid portion of each glioma and obtained pixel values [4], [5], [7], [9], [10], [25]–[27]. However, for high-grade-gliomas, which are inherently heterogeneous with various tumor grades in a single tumor [6], placing ROIs on the most malignant portion of the tumor is completely user-dependent. Thus, the potential advantage of the histogram method is that obtaining the total voxel values of a tumor would provide data that are more objective and that exhibit less interobserver variability for identifying the most malignant portion in a single tumor [6], [8]. Emblem et al. [6] concluded that the observers in their study were not able to consistently identify the most malignant tumor region with the conventional ROI method (the so-called hot-spot method).

For glioma grading using the cumulative histogram method, Kang et al. [8] reported that the 5^th^ percentile value (C5) of the cumulative ADC histogram was the most promising parameter for differentiating high- from low-grade gliomas. However, cumulative percentile values were evaluated for only the 5^th^, 50^th^ and 75^th^ percentiles in the study. It is possible that the lower-end percentile values below the 5^th^ percentile on the cumulative histogram exhibit higher diagnostic accuracy. In this context, for the PWI, we evaluated the percentile values continuously from the 70^th^ to the 100^th^ percentile of the cumulative nCBV histogram to determine the optimum percentile value for the glioma grading. The 70^th^ percentile as the lower limit was chosen empirically to sufficiently cover more than the upper quarter. Subsequently, the nCBV C99 exhibited the highest AUC on the ROC curve analysis.

In terms of nCBV histogram parameters other than the cumulative histogram parameters for the differentiation of tumor grades, Emblem et al. [6] reported that glioma grading based on a histogram analysis of nCBV heterogeneity resulted in higher interobserver agreement, sensitivity and equal specificity compared with the conventional ROI method. According to that study, the maximum peak height of the nCBV histogram was lower and exhibited a wider distribution of pixel values in high-grade gliomas, which represented the tumor heterogeneity in high-grade tumors [6]. Law et al. [11] demonstrated that nCBV histogram parameters such as the SD, SD of the top 50% of nCBV values (SD_50_) and mean of the top 25% of nCBV values (Mean_25_) were useful for glioma grading. However, only a single section from a perfusion dataset was used to determine the nCBV in that study. Young et al. [13] compared the three different histogram methods of tumoral (ROI drawn on a single slice), peri-tumoral (defined by a semi-automated process as the area around and not including the manually drawn tumoral ROI) and total tumoral (all acquired perfusion images covering the tumor without any segmentation) histograms and found that the histogram parameters such as the tumoral nCBV SD, peri-tumoral nCBV SD_25_ and total tumoral nCBV SD_50_ displayed the strongest correlations with the glioma grade. Recently, Garzon et al. [16] performed a retrospective analysis to investigate multiple MR-derived image features, including histogram parameters, with respect to diagnostic accuracy in tumor grading and found that the presence/absence of enhancement paired with kurtosis of the first moment of the first-pass curve was the feature combination that best predicted tumor grade.

Using the same software package for the image post-processing (nordicICE; NordicNeuroLab AS, Bergen, Norway) as in the previous studies [6], [12], [16], our study focused on the percentile values on a cumulative histogram as well as the peak height of a histogram. Similar to the previous studies, we revealed that assessing the entire tumor voxel values of the nCBV was promising for glioma grading with high diagnostic accuracy. The nCBV C99 exhibited sensitivity, specificity and diagnostic accuracy values of 85.2%, 100% and 87.3%, respectively. The peak height of the histogram exhibited sensitivity, specificity and diagnostic accuracy values of 83.3%, 100% and 85.7%, respectively. Emblem at al. study [6] reported sensitivity and specificity values of 90% and 83% for peak height, which were similar to those in our study. The nCBV C99 in the present study displayed higher diagnostic accuracies for differentiating high- from low-grade gliomas and grade IV from III gliomas than the peak height; however, there were no significant differences in diagnostic accuracies between the two parameters after the Bonferroni correction.

In previous studies using the conventional ROI method for differentiating high- from low-grade gliomas, researchers have suggested several different cutoff values for the rCBV. Lev and Rosen [28] used an rCBV cutoff value of 1.5 with a sensitivity and specificity of 100% and 69%. Law et al. [29] defined a threshold value of 1.75 with a sensitivity and specificity of 95% and 57.5%. Shin et al. [30] reported a sensitivity and specificity of 91% and 83% with a cutoff value of 2.9. Recently, Hilario et al. [7] reported a sensitivity and specificity of 94.4% and 50% with a cutoff value of 1.74. In these studies, the ROI was placed in the region of maximal perfusion, which was determined visually using an rCBV map and the mean rCBV of the ROI was obtained. For this reason, the cutoff value of nCBV C99 in our study (4.681) was much higher than previously used threshold values. It is reasonable that the single-voxel C99 values of gliomas from low- to high-grades are higher than the mean rCBV of the maximal perfusion regions obtained with the conventional ROI method. Interestingly, the mean nCBV cutoff value of 1.786 in our study was comparable to past study results, although it displayed a lower diagnostic accuracy than nCBV C99.

We found that the advantage of the cumulative nCBV histogram method in glioma grading is that it can provide high diagnostic accuracy in differentiating grade IV from grade III gliomas, as well as high- from low-grade gliomas. Hilario et al. [7] previously reported significant differences in rCBV values between grade III and IV gliomas and Emblem et al. [6] suggested the potential advantage of the histogram method in differentiating between grade III and IV gliomas. However, diagnostic accuracies were not reported in those studies. We revealed that grade IV gliomas could be differentiated from grade III gliomas with a sensitivity and specificity of 84.2% and 100% when the nCBV C99 cutoff value of 6.335 was used. Thus, we suggest a potential glioma grading schema using two nCBV C99 cutoff values for separating the three different WHO gliomas grades from II to IV (Fig. 6). By using this grading schema, we could diagnose grade II gliomas with 100% (9/9) diagnostic accuracy, grade III gliomas with 50% (8/16) diagnostic accuracy and grade IV glioblastomas with 84.2% (32/38) diagnostic accuracy. When the cutoff values were applied to the test set of nine patients, we could diagnose grade II gliomas with 100% (2/2) diagnostic accuracy, grade III gliomas with 33.3% (1/3) diagnostic accuracy and grade IV glioblastomas with 75% (3/4) diagnostic accuracy (Table 5). We admit that the separation of grade III gliomas from other tumors was not satisfactory with the suggested cutoff values; however, the value of this grading schema is that we could accurately differentiate low- from high-grade-gliomas and grade IV from grade II and III gliomas. One plausible cause of the high diagnostic accuracy in the present study is that gliomas with oligodendroglial components were excluded. Oligodendroglial tumors are known to exhibit a relatively high tumor blood volume [31] and would thus increase the mean maximum nCBV in grade II and III gliomas.

Apart from the intrinsic limits of any retrospective study, several other limitations of our study should be mentioned. First, only a small number of low-grade gliomas (n = 9) was included; however, it is well known that low-grade gliomas account for 10–15% of all adult primary intracranial tumors [32], which is very similar to our study setting. Future prospective studies with a larger number of patients would be necessary to verify our cumulative histogram parameter findings for glioma grading. Second, tumor boundary was defined with reference to high signal intensity on T2W images and tumor infiltration as well as peri-tumoral edema was included in the ROIs. However, the differentiation between these two components is impossible in the imaging studies. Third, the nCBV measurement variability, such as the intraobserver or interobserver variability, was not calculated in the study. The histogram analysis method has been found to be less observer-dependent [6], [11] and the ROI in this study included the whole tumor volume and was not placed on a selected portion of the tumor subjectively. However, the measurement variability in image interpretation is increasingly considered an important performance metric of radiologic research [33]. The nCBV measurement variability using ‘nordicICE’ requires a comprehensive validation study in the future. Fourth, the normal-appearing white matter as a reference region for the calculation of nCBV may potentially be affected by, for example, radiation therapy and tumor invasion [34]. However, we excluded the patient who underwent chemoradiotherapy prior to the imaging and tumor invasion was evaluated on the conventional MR sequences as best we could manage. Fifth, histologic diagnoses of the 16 patients out of the 63 total enrolled patients were based on stereotactic biopsies. As high-grade glioma exhibits a continuum of histologic features from grade II to IV [7], there is the possibility of histologic misdiagnosis due to sampling error. Sixth, the exclusion of the oligodendroglial tumors is potentially controversial. Although Cha et al. [31] reported that oligodendroglial components increase tumor blood volume, the peak height of the histogram could be used to differentiate high- from low-grade gliomas even in the tumors with oligodendroglial components [12]. As the main purpose of the present study was to evaluate cumulative histogram analysis for pure astrocytic tumor grading, future studies examining oligodendroglial tumors are warranted.

In conclusion, this study reveals that the nCBV C99 could be used for distinguishing high- from low-grade gliomas and grade IV from III gliomas with diagnostic accuracies of 87.3% and 88.9%, respectively. We suggest a potential glioma grading schema using two nCBV C99 cutoff values for separating the three different WHO glioma grades.
